# Potential impact of COVID-19 related unemployment on increased cardiovascular disease in a high-income country: Modeling health loss, cost and equity

**DOI:** 10.1371/journal.pone.0246053

**Published:** 2021-05-27

**Authors:** Nhung Nghiem, Nick Wilson

**Affiliations:** Department of Public Health, University of Otago, Wellington, New Zealand; University of Western Australia, AUSTRALIA

## Abstract

**Background:**

Cardiovascular disease (CVD) is a leading cause of health loss and health sector economic burdens in high-income countries. Unemployment is associated with increased risk of CVD, and so there is concern that the economic downturn associated with the COVID-19 pandemic will increase the CVD burden.

**Aims:**

This modeling study aimed to quantify potential health loss, health cost burden and health inequities among people with CVD due to additional unemployment caused by COVID-19 pandemic-related economic disruption in one high-income country: New Zealand (NZ).

**Methods:**

We adapted an established and validated multi-state life-table model for CVD in the national NZ population. We modeled indirect effects (ie, higher CVD incidence due to high unemployment rates) for various scenarios of pandemic-related unemployment projections from the NZ Treasury.

**Results:**

We estimated the potential CVD-related heath loss in NZ to range from 23,300 to 36,900 health-adjusted life years (HALYs) for the different unemployment scenarios. Health inequities would be increased with the per capita health loss for Māori (Indigenous population) estimated to be 3.7 times greater than for non-Māori (49.9 vs 13.5 HALYs lost per 1000 people). The estimated additional health system costs ranged between (NZ$303 million [m] to 503m in 2019 values; or US$209m to 346m).

**Conclusions and policy implications:**

Unemployment due to the COVID-19 pandemic could cause significant health loss, increase health inequities from CVD, and impose additional health system costs in this high-income country. Prevention measures should be considered by governments to reduce this risk, including additional job creation programs and measures directed towards the primary prevention of CVD.

## Introduction

The COVID-19 pandemic had infected 65 million people and caused over 1.5 million deaths globally at the time of writing (early December 2020) [1, 2]. Unfortunately at this time, pandemic spread was still accelerating and so its final impact before widespread vaccination is likely to be substantially greater.

Globally, this pandemic has disrupted international travel and domestic economies (via depressed consumer mobility due to fear of infection and also the use of “lockdowns” that close some workplaces and social settings). As such it has depressed gross domestic product (GDP) with this reduction for OECD countries being by -10.5% in the second quarter in 2020 [3], and with an increase in unemployment by an absolute increment of 3.2% (8.6% in the second quarter vs 5.4% in the first quarter) [4] for these countries [5–7]. There is established evidence that economic recessions and depressions can increase poor health [8, 9]. This includes cardiovascular disease (CVD) [8–10] as per our recent review of this topic [11]. In particular, one meta-analysis [9], which included 174,438 participants with a mean follow-up of 9.7 years and 1892 incident cases of CVD from 13 cohort studies, reported that increased job insecurity was associated with increased CVD incidence. Also, there is evidence that people experiencing economic hardship are at higher risk of CVD mortality [8, 9, 12]. Stress and loneliness (eg, potentially exacerbated by pandemic-related lockdowns) are also known risk factors for CVD onset [13–15].

According to the Global Burden of Disease Study 2017, CVD is still the leading cause of death in the world at an estimated 17.8 million deaths [16]. In New Zealand (NZ), CVD makes up around 14% of all health loss nationally, and is responsible for a third of the total number of deaths annually [17]. CVD is a particularly important contributor to health loss for the Indigenous Māori population [17, 18] and it contributes to health inequities in NZ in terms of both ethnicity and socioeconomic position [19–21].

Although NZ has had a relatively successful health sector response to the COVID-19 pandemic (with an elimination strategy [22]), the economic impact of the response (via lockdowns, lost revenue from international tourism and international students) has been severe [23, 24].

This study therefore aimed to estimate via modeling, the health loss, health cost burden and health inequities due to CVD onset as a result of unemployment caused by the COVID-19 pandemic in NZ [25, 26].

## Methods

We adapted an established and validated multi-state life-table model for CVD for the national NZ population [27–29]. We modeled only indirect effects (ie, higher CVD incidence due to high unemployment rates) arising from COVID-19 pandemic-related unemployment. Direct effects of the COVID-19 pandemic onto CVD were not considered since the disease burden (only 25 deaths as of 15 December 2020) has been relatively low in NZ prior to the elimination of community transmission. We used various NZ Treasury scenarios of the COVID-19 pandemic’s impact on unemployment projections in NZ [23, 30]. The timeline for the pandemic impact on unemployment was for five years as per the Treasury projections, but a lifetime horizon was used for measuring benefits and costs (a five-year horizon was also implemented in a scenario analysis).

### Unemployment scenarios due to the COVID-19 pandemic and the response in NZ

The NZ Government stated that “the COVID-19 pandemic is a ‘once in a century’ public health shock that is also having a profound impact on economic and financial systems around the world and in NZ” [30]. The Government also suggested that the impact of COVID-19 and related response measures on the NZ economy is highly uncertain. So the NZ Treasury presented four different economic scenarios based on health response measures and on key economic assumptions, including the reduction in production output in NZ, the Government’s support for households and businesses, and the world’s real production outputs. Detailed scenarios including the baseline unemployment projection before COVID-19, the base case and alternative scenarios (ie, earlier recovery in services exports, extended border controls, and resurgence in community transmission) are presented in Table 1. The key estimates were that the peak of unemployment rates would vary from 7.7% in the most relaxed response, to 9% in the most restrictive one (Table 1). In all of the scenarios, however, the unemployment rates were still higher than that in the baseline scenario (had there never been a pandemic) which was around 4% after five years (in 2024).

**Table 1:** Projected unemployment scenarios in NZ as a result of the COVID-19 pandemic and response to it (extracted from a NZ Treasury Report) [23, 30].

Scenario description	Projected unemployment rates (2020–2024)	Assumptions around border restrictions and exports of services*
Baseline	The unemployment rate as per 2019 (4%)	Business–as–usual as if there was no COVID-19 pandemic.
Base case**	The unemployment rate is 6.9% on average over the forecast period. At the end of the forecast, the unemployment rate is 5.3%. (5.3***, 7.7, 7.6, 6.6, 5.3)	Border restrictions are partially eased on July 2021 and fully removed on January 2022. Real services exports are 13% lower at the end of the forecasting period than in the December 2019 quarter.
Earlier recovery in exports of services	The unemployment rate is 6.2% on average over the forecast period and is 4.8% at the end of the forecast. (5.3, 7.7, 6.4, 5.5, 4.8)	Border restrictions are unchanged from the main forecast, but exports of services recover more strongly. Real services exports are 4% lower at the end of the forecasting period than in the December 2019 quarter.
Extended border controls	The unemployment rate is 7.5% on average over the forecast period and is 6.6% at the end of the forecast. (5.3***, 7.7, 8.1, 7.6, 6.6)	Border restrictions are partially eased on July 2021 but there is no further substantive easing. Real services exports are 35% lower at the end of the forecasting period than in the December 2019 quarter.
Resurgence in community transmission	The unemployment rate is 7.8% on average over the forecast period and is 5.8% at the end of the forecast. (5.3***, 9.0, 8.5, 7.3, 5.8)	Border restrictions and real services exports are unchanged from the main forecast; however, potential output is 0.6% lower, and real GDP is 1% lower, at the end of the forecast period.

Notes:

* Services exports such as international education and international tourism in NZ.

** The base case and alternative scenarios were extracted from Fig 1.20 in an Updated Treasury Report (16 September 2020).

*** Actual unemployment rate in the September quarter in 2020.

### Calibration of the changes in unemployment rates due to the COVID-19 pandemic by age, sex and ethnicity

In the NZ Treasury Reports [23, 30], projected unemployment rates were not disaggregated by age, sex and ethnicity. We therefore calibrated the projected changes in unemployment rates annually between 2020–2024, so that it could be used directly in our model as follows.

#### Data

We averaged the unemployment rates by ethnicity (i.e, Māori and non-Māori) over 2010–2014 [31, 32]. We also averaged the unemployment rates by sex and by age over 2010–2014. Of note is that these rates were for people aged 15–64 years old in NZ. We used real population by age, sex and ethnicity from Census 2013 to calibrate the unemployment rates [33].

#### Assumption

We assumed that the relative rate of unemployment rates by age, sex and ethnicity before the COVID-19 pandemic held. That meant that:

we=UMUNM=dUMdUNM (1)

ws=UfUm=dUfdUm (2)

wa35_44=Ua35-44Ua45-54=dUa35-44dUa45-54 (3)

Where *U* is unemployment rate, *dU* is the absolute change in unemployment rate, *w*_*e*_ is the weight of unemployment rate for Māori over non-Māori, *w*_*s*_ is the weight of unemployment rate for women over men, and *w*_a3544_ is the weight of unemployment rate for people aged 35–44 over the reference age-group which is people aged 45–54 years.

#### Algorithm

From the assumptions in Eqs (1) to (3), we derived the absolute changes in unemployment rates by ethnicity, sex and 10-year age-group, in sequence, as follows:

$${dU}_{M}=\frac{dU\times U_{M}}{{pop}_{M}\times U_{M}+{pop}_{NM}\times U_{NM}}$$ (4)

$${dU}_{f}=\frac{dU\times U_{f}}{{pop}_{f}\times U_{f}+{pop}_{m}\times U_{m}}$$ (5)

$${dU}_{a35-44}=\frac{dU\times U_{a35-44}}{{pop}_{a35-44}\times U_{a35-44}+{pop}_{a45-54}\times U_{a45-54}+{pop}_{55-64}\times U_{a55-64}+{pop}_{a15-24}\times U_{a15-24}+{pop}_{a25-34}\times U_{a25-34}}$$ (6)

Where *pop*_*_M*_ is the real Māori population in 2013, *pop*_*_ a35-44*_ is the real population aged 35–44 years, and so on. Of note is that if any of the assumptions in Eqs (1)–(3) are altered, the absolute changes derived in Eqs (4)–(6) are changed accordingly. Furthermore, we assumed sex and age patterns (Eqs (5)–(6)) followed that for the general population, not by each ethnicity. This involves a slight simplification since there are minor differences in age structure by ethnicity within the studied age-groups, eg, the Māori population has a younger age-group. But as young people have higher unemployment rates, by using the age structure for the general population, our results were conservative towards smaller changes in absolute unemployment rates.

#### Output

The output of the calibration was absolute changes in unemployment rates (as projected by the Treasury [23, 30]) by ethnicity (Māori/non-Māori), sex and 10-year age-group (35–64 years old) for each unemployment scenario annually (2020–2024).

### Associations between unemployment, all-cause mortality and CVD incidence

In this study, we modeled health burdens from pandemic-induced unemployment in terms of CVD morbidity and mortality. We focused on only modeling the impact of unemployment on CVD incidence, and ignored other economic impacts arising from the reduction in real GDP.

For the impact of unemployment on CVD incidence, we used the results from a large multi-country study by Stuckler et al in 2009 [10]. We considered this work to be the most robust of all the studies we identified in a recent review [11]. This work reported a 0.85% relative increase in CVD incidence for a 1% relative increase in the unemployment rate in middle-aged men (or a relative risk (RR) of 1.0085 for CVD incidence (including both coronary heart disease [CHD] and stroke)). While Stuckler et al 2009 considered only CVD mortality—for modeling purposes we assumed that this impact was entirely due to increased CVD incidence and not any additional deterioration in CVD case-fatality risk (where we assumed no change in the pre-existing downward trend (Table 2)). There will be some competing mortality reasons why a 0.85% increase in incidence doesn’t perfectly align with a 0.85% increase in CVD mortality—and so we reported how the model output actually responds to the 0.85% incidence increase.

**Table 2:** Associations between unemployment and CVD incidence used in this modeling study adapted from the literature (Stuckler et al 2009 [10]).

Population group	Association of a 1% relative increase in unemployment on CVD mortality percentage changes (% and (CIs)) as per Stuckler et al 2009	Change in annual CVD incidence (RR(CIs)) (converted to so as to use in our modeling work)
Women, aged 35–44	-0.14 (-1.95–1.67)	-1.0014 (-1.0195–1.0167)
45–59	0.10 (-1.09–1.30)	1.0010 (-1.0109–1.0130)
60–64	0.23 (-0.22–0.68)	1.0023 (-1.0022–1.0068)
Men, aged 35–44	0.85 (0.06–1.64)	1.0085 (1.0006–1.0164)
45–59	0.48 (-0.06–1.02)	1.0048 (-1.0006–1.0102)
60–64	0.38 (-0.16–0.91)	1.0038 (-1.0016–1.0091)

We also assumed that the effect size in CVD incidence increased linearly with an increase in unemployment rate, ie, a 2% increase in relative unemployment rate translated to 1.7% relative increase in CVD incidence rate with a RR of 1.0085. The assumption of linearity is the most parsimonious assumption—especially for these relatively small changes. But it is plausible that large increases in unemployment would have normalizing effects and perhaps reduce the impact on CVD (eg, if a large increase in unemployment triggered a disproportionate government response such as a major expansion of welfare provisions).

### Key model parameters: Epidemiology, economic and unemployment

We presented all epidemiology and economic key parameters as per the established and validated multi-state life-table BODE^3^ model for CVD for the national NZ population in Table 3 [27–29].

**Table 3:** Key epidemiology and economic parameters for the established BODE^3^ CVD model [27–29].

Parameters	Value	Heterogeneity	Uncertainty
All-cause mortality and time trends	As per the BODE^3^ CVD model [27–29]. Trends for all-cause mortality were made consistent with long-run mortality trends for NZ (annual 2.25% mortality decline for Māori and 1.75% per annum for non-Māori). Trends were modeled out to 2026 (ie, for 15 years), with no subsequent decline for both ethnic groupings thereafter.	By ethnicity (Māori/non-Māori), sex and age	No
Baseline CVD incidence/morbidity/mortality and time trends	As per the BODE^3^ CVD model [27–29]. As per the NZ Burden of Disease Study (NZBDS) [34] we assumed a continued decline in incidence rates for both CHD and stroke of 2.0% annually, and also a 2.0% annual reduction in case fatality so that it reflects improved treatment and management.	By ethnicity (Māori/non-Māori), sex and age	Uncertainty: ±5% SD, log-normal distribution.
Background health system costs for all citizens (adjusted for CHD and stroke costs)	As per BODE^3^ costing methods [35]	By sex and age	Uncertainty: ±10% SD, log-normal distribution.
Unemployment duration	5 years as per the NZ Treasury report [23].	No	No, but we conducted a sensitivity analysis whereby unemployment persists for 10 years.
Unemployment rates	As per the NZ Treasury’s report over 2020–2024 (Table 1)	By ethnicity (Māori/non-Māori), sex and age	All unemployment rates followed a log-normal distribution with an assumed 20% standard deviation (due to all health responses and economic outlooks being highly uncertain).
Discount rate	3% for both health losses and costs as per standard BODE^3^ methods	No	No
Perspective	Health system as per standard BODE^3^ methods	No	No
Time horizon	Lifetime (until died or 110 years old)	No	No

**Fig 1:**
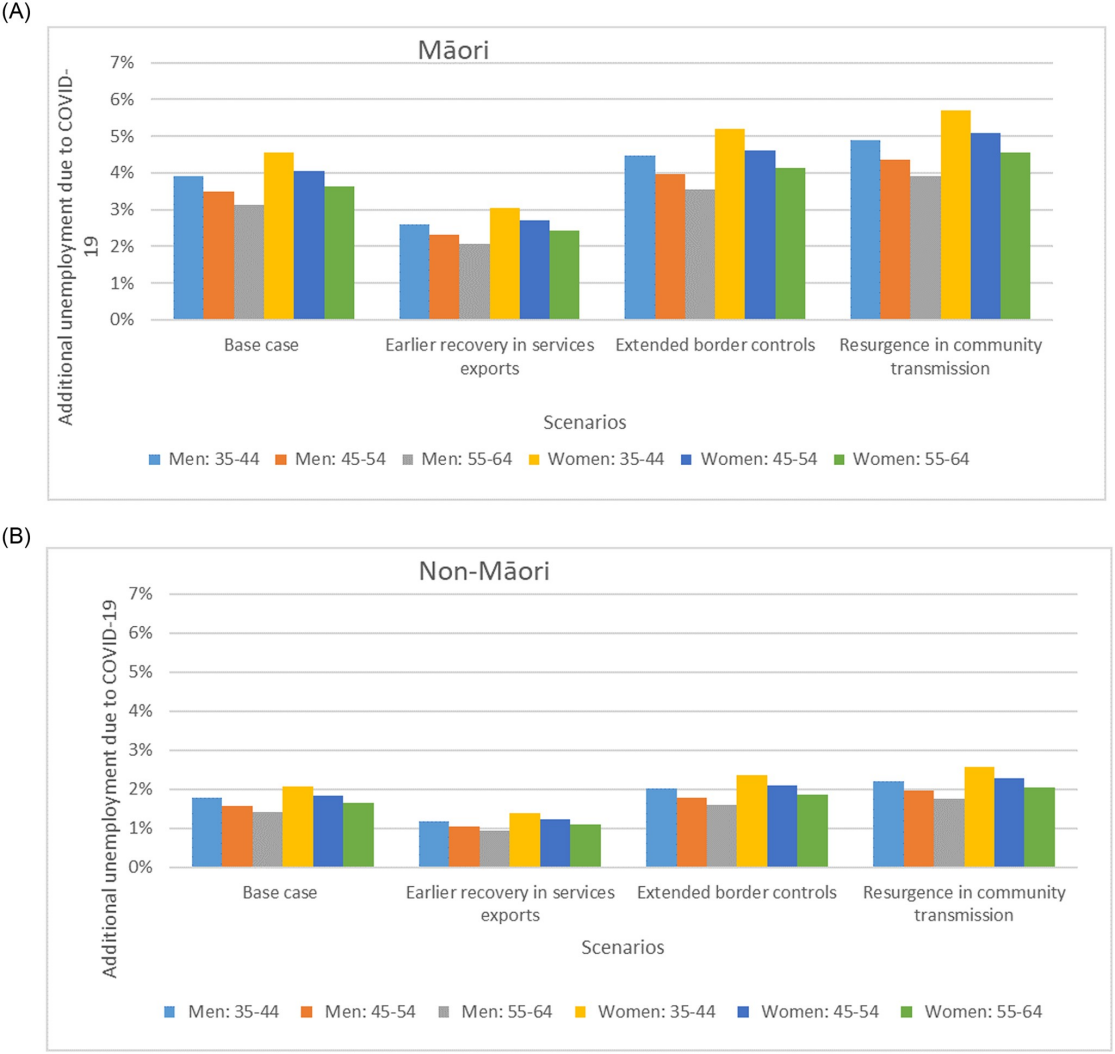
Scenarios around the COVID-19 pandemic related additional absolute unemployment rates in 2021 used in the BODE^3^ CVD model by age, sex and ethnicity in NZ^a,b,c,d^. ^a^ These unemployment rates were the differences in the projected unemployment rates between having the COVID-19 pandemic (Scenarios in Table 1) and having not had the COVID-19 pandemic (Baseline Scenario in Table 1). They were also adjusted by the unemployment rates by age, sex and ethnicity in 2010–2014 reported by the NZ Government. ^b^ All unemployment rates followed a log-normal distribution with an assumed 20% standard deviation (due to all health responses and economic outlooks being highly uncertain). ^c^ We only extracted unemployment rates for these age-groups as they are in typical working age-groups, and are potentially impacted by the CVD burden. But we acknowledge this limitation further in the Discussion. ^d^ The age-groups in this Table do not exactly match the ones in Table 2, but we used the closest age-group for RR and additional unemployment rate in our model. Note that in 2021, unemployment rates are similar across scenarios (see Table 1). Furthermore, in the Treasury economic models, the projected unemployment levels take into account the impact from GDP and other services such as impacts from the continued loss of international students and international tourists.

We calculated unemployment rates by age, sex and ethnicity (Fig 1) used in the BODE^3^ CVD model for each economic scenario listed in Table 1. These were the differences in the projected unemployment rates between the pre-COVID-19 baseline and after the appearance of the COVID-19 pandemic (Baseline Scenario in Table 1). All unemployment rates followed a log-normal distribution with an assumed 20% standard deviation (due to all health responses and economic outlooks being highly uncertain).

Of note is that due to the design of the BODE^3^ CVD model (ie, modelling the whole NZ population alive in 2011 throughout their life or until 110 years), we modeled the unemployment impacts due to the COVID-19 pandemic as if they were happening in the 2011–2015 period.

### Additional scenarios and sensitivity analysis

Further to four economic scenarios as mentioned above (eg, earlier recovery in exports of services, and extended border controls), we also modeled the following sensitivity analyses:

1. Māori CVD epidemiology parameters as per non-Māori to avoid penalizing Māori due to lower life expectancy and higher comorbidity,
2. Unemployment persists for longer (ie, 10 years),
3. No reduction in CVD trend over time (eg, on the assumption that the long-term downward trend in CVD is halted by the obesity epidemic—as seen in some high-income countries) [36].
4. Zero percent discount rate, and
5. Six percent discount rate.

### Ethics approval

Approval for use of anonymized administrative data as part of the BODE^3^ Programme has been granted by the Health and Disability Ethics Committees (reference number H13/049).

## Results

Table 4 shows the estimated CVD-related health loss (in HALYs) for the base case (most likely scenario) (3% discount rate for the remaining life of the NZ population alive in 2011) for various COVID-19 pandemic induced unemployment scenarios (base case, early recovery and extended border control). The health loss for the base-case was estimated at -30,300 HALYs (uncertainty interval: -66,700 to 4,100). However, only 10% of the loss (-3,010 HALYs) was accumulated in the first five years and more than 50% (-17,770 HALYs) in the 20 years in the future. The estimated CVD-related heath loss in NZ ranged from 23,300 (early recovery in export and services) to 36,900 lost HALYs (resurgence in community transmission) for the different unemployment scenarios. Of which, health loss for Māori ranged between -7,700 HALYs and -13,000, and for non-Māori from -15,700 to -26,600.

**Table 4:** Health loss (in HALYs) results for the base case (most likely unemployment scenario) (3% discount rate for the remaining life of the NZ population alive in 2011) for various COVID-19 pandemic-induced unemployment scenarios from the NZ Treasury.

Population groups	Change in HALYs				Change in HALYs per 1000 capita				Equity: Māori/Non-Māori Loss in HALYs per capita
	Base case	Early recovery in trade and services	Extended border control	Resurgence in community transmission	Base case	Early recovery in trade and services	Extended border control	Resurgence in community transmission	Base case
All ethnic groups, both sexes, all age-groups	-30,300 (-66,700; 4,100)	-23,300	-36,900	-39,600	-17.7	-13.6	-21.5	-23.1	…
5 years into future	-3,010 (-6,910; 440)	-2,450	-3,440	-3,940	-1.8	-1.4	-2.0	-2.3	…
10 years into future	-7,950 (-17,580; 850)	-6,250	-9,430	-10,370	-4.6	-3.6	-5.5	-6.0	…
20 years into future	-17,770 (-38,110; 1,520)	-13,800	-21,400	-23,200	-10.4	-8.0	-12.5	-13.5	…
Māori, both sexes, all age-groups	-9,910 (-24,750; 4,730)	-7,700	-12,200	-13,000	-49.9	-38.5	-61.4	-65.5	3.7
Non-Māori, both sexes, all age-groups	-20,440 (-42,040; 170)	-15,700	-24,700	-26,600	-13.5	-10.3	-16.3	-17.5	…
Māori Men, all age-groups	-8,930 (-15,950; -2,830)	-6,820	-10,770	-11,540	-95.9	-73.2	-115.7	-124.0	3.9
Non-Māori Men, all age-groups	-18,170 (-32,180; -5,220)	-13,900	-21,800	-23,500	-24.7	-18.9	-29.6	-31.9	…
Māori Women, all age-groups	-980 (-14,640; 12,570)	-830	-1,440	-1,470	-9.3	-7.9	-13.6	-13.9	3.2
Non-Māori Women, all age-groups	-2,270 (-18,600; 14,050)	-1,790	-2,900	-3,070	-2.9	-2.3	-3.7	-3.9	…
Māori Men 35–44	-3,570 (-6,850; -710)	-2,720	-4,330	-4,630	-93.2	-71.0	-113.1	-120.9	4.5
Māori Men 45–54	-3,490 (-7,460; 130)	-2,610	-4,270	-4,490	-103.3	-77.4	-126.3	-132.8	4.0
Māori Men 55–64	-1,870 (-3,690; -190)	-1,480	-2,170	-2,430	-89.0	-70.7	-103.3	-115.7	3.2
Non-Māori Men 35–44	-5,160 (-9,860; -1,140)	-3,910	-6,260	-6,680	-20.7	-15.7	-25.1	-26.8	…
Non-Māori Men 45–54	-6,850 (-14,810; 260)	-5,090	-8,410	-8,810	-25.8	-19.1	-31.6	-33.1	…
Non-Māori Men 55–64	-6,160 (-12,480; -320)	-4,880	-7,130	-8,010	-27.8	-22.1	-32.2	-36.2	…
Māori Women 35–44	470 (-6,100; 6,920)	328	421	512	10.7	7.4	9.5	11.6	6.9
Māori Women 45–54	-610 (-8,790; 7,570)	-514	-843	-887	-16.0	-13.5	-22.1	-23.2	6.0
Māori Women 55–64	-840 (-3,510; 1,870)	-648	-1,018	-1,095	-36.2	-27.9	-43.9	-47.2	4.3
Non-Māori Women 35–44	420 (-5,510; 6,250)	301	384	469	1.5	1.1	1.4	1.7	…
Non-Māori Women 45–54	-740 (-10,350; 8,750)	-590	-985	-1,027	-2.6	-2.1	-3.5	-3.7	…
Non-Māori Women 55–64	-1,940 (-7,450; 3,520)	-1,500	-2,300	-2,510	-8.5	-6.5	-10.1	-11.0	…

**Notes**: *All ages means aged 35–64 years. The equity ratio for Māori/Non-Māori HALYs lost per capita was very similar for all scenarios.

For the base case (best-estimate) scenario, health inequities were estimated to increase. That is for Māori (Indigenous population) the per capita health loss was 3.7 times greater than for non-Māori (49.9 vs 13.5 HALYs lost per 1000 people). Māori men suffered the most health loss per capita (95.9 HALYs per 1000 people); however, the worst inequity impacts between Māori and non-Māori were seen in women aged 45–54 years across all scenarios (six fold difference in per capita health loss). Inequities were exacerbated much more than the sum of existing inequities in CVD burden and unemployment, ie, in some groups, the health loss from CVD burden for Māori was increased to six times higher than that of non-Māori (compared to a double unemployment rate or background CVD burden). The overall pattern of inequity by ethnicity, age and sex was similar for the various modeled economic scenarios.

Table 5 presents the additional health costs estimated to be incurred to the NZ health system due to the higher CVD burden. The estimates ranged between NZ$276 million (m) to 458m in 2011 values (NZ$303m to 503m or US$209m to 346m in 2019 values) for the various unemployment scenarios. Similar to the health burden, 10% of the additional health cost was accumulated in the first five years and more than 60% in the 20 years in the future.

**Table 5:** Additional health system costs for the base case (most likely unemployment scenario) (3% discount rate for the remaining life of the NZ population alive in 2011).

Groups	Additional health system costs (NZ$ million [m])				Additional health system costs (NZ$ m) per 1000 capita			
	Base case	Early recovery in trade and services	Extended border control	Resurgence in community transmission	Base case	Early recovery in trade and services	Extended border control	Resurgence in community transmission
All ethnic groups, both sexes, all age-groups	354 (-78; 824)	276	429	458.0	0.21	0.16	0.25	0.27
5 years into future	234 (-6; 501)	187	276	301.0	0.14	0.11	0.16	0.18
10 years into future	324 (-26; 703)	254	388	418.0	0.19	0.15	0.23	0.24
20 years into future	402 (-63; 902)	312	487	520.0	0.23	0.18	0.28	0.30

The CVD-related health loss, inequities and health costs were similar in terms of relative patterns across scenario analyses and sensitivity analyses (S1–S5 Tables). The equity scenario (Scenario #A, S1 Table) suggested more health loss (-10,800 vs -9,910 HALYs in the base case) and more inequity (up to a ratio of Māori/non-Māori lost HALYs per capita of 7.1), if Māori CVD epidemiology parameters were assumed to be as per non-Māori (ie, same risk of CVD mortality and morbidity). Unemployment persisting for 10 years (#B) suggested the most health loss (-57,100 HALYs), and 6% discount rates (#E) resulted in the smallest estimated health loss (-18,500 HALYs).

## Discussion and conclusions

### Main results and interpretation

In this modeling study, we estimated the CVD-related heath loss in NZ to range from 23,300 to 36,900 HALYs for the different unemployment scenarios arising from the COVID-19 pandemic. For the base case (best-estimate) scenario, health inequities were estimated to increase, with a 3.7 times greater per capita health loss for Māori. While Māori men suffered the most health loss per capita, the worst inequity impacts between Māori and non-Māori were estimated to be in women aged 45–54 years across all scenarios (six fold difference). Inequities were exacerbated much more than the sum of existing inequities in CVD burden and unemployment, ie, in some groups, the Māori per capita health loss from CVD was increased to six times higher than that for non-Māori (compared to a double unemployment rate or background CVD burden). The additional health costs due to the higher CVD burden were also substantial (at NZ$276m to 458m in 2011 values) for the various unemployment scenarios.

### Study strengths

The first major strength of this study was the use of a validated disease model that has been previously applied in the NZ context. Second, we followed economic scenarios projected by the NZ Treasury with detailed assumptions on border controls, international tourism and education for NZ. Third, as the COVID-19 pandemic response in NZ was among the strictest [37] and one of the most successful internationally as of December 2020 [38], our analysis reflects a pandemic-related impact that is relatively independent of the direct disease impacts (ie, it reflects unemployment from the indirect impacts of the NZ response and international impacts impacting on trade). Finally, our study benefited from detailed unemployment data by age, sex and ethnicity in NZ.

### Study limitations

We did not consider that all-cause mortality and CVD mortality could also change due to behavioral changes vs supply changes (tobacco, fast food, alcohol, and physical activity levels) as a result of the COVID-19 pandemic restrictions. For example, if a pandemic-induced recession lowers the affordability of tobacco and alcohol—then there might be non-linear social contagion impacts as whole cohorts of people reduce consumption of these products in social settings (eg, going out less to bars and restaurants). But we had inadequate data on such impacts to consider these in this research. Also, if increased unemployment-induced poverty resulted in less affordable tobacco and less expenditure on junk food (generally more expensive than home-cooked food in NZ [39]), then those factors would tend to reduce CVD. Finally, we ignored people aged 64+ who are in retirement age (although around 20% of this group are still in formal employment in NZ).

As mentioned in the Methods, due to the design of the BODE^3^ CVD model, we modeled the unemployment impacts due to the COVID-19 pandemic as if they were happening in the 2011–2015 period. This is likely to slightly over estimate the lost HALYs due to slightly higher CVD prevalence rates in 2011, assuming a downward trend in CVD prevalence over time. Furthermore, Stuckler et al 2009 considered only CVD mortality changes due to unemployment—for modeling purposes we assumed that this impact was entirely due to increased CVD incidence and not any additional deterioration in CVD case-fatality risk. Also while it is possible that there is an effect of recessions on poorer health care delivery care (that might increase case fatality risk in those with CVD), in the absence of data we assumed no such additional impact.

### Potential policy implications

This modeling work suggests that unemployment due to the COVID-19 pandemic could potentially cause significant health loss and health costs from CVD in NZ. Furthermore, these pandemic impacts could exacerbate the health inequities in CVD for Māori, that are already due to higher background risks of CVD. Additional prevention measures should be considered by central and local governments to reduce the risk of increased unemployment, including via additional job creation programs. Indeed the NZ Government has invested to some extent in such programs in the COVID-19 recovery period. The Government could also increase interventions aimed at the primary prevention of CVD eg, enhanced progress towards a smokefree country and reducing the hazardous processed food environment, as we discuss elsewhere [11].

### Further research work

Further work could be done on modeling the health burden, health inequity, and health cost impacts of COVID-19-related unemployment. Since our study used the NZ Treasury’s economic scenario projections, not the actual increased unemployment, this work could be repeated with real world estimates of unemployment. For example, this could use unemployment data after six months of the Australia-NZ quarantine-free travel zone operating (which is expected to help with tourism sector recovery in NZ and potentially better represent a new-normal, at least during the 2021 year). It may also be worth modeling the impact of unemployment reduction interventions. Examples could include the impact of various job creation programs. In addition, a modeled intervention could assume that the NZ Government could target job creation for Māori so that the absolute increases in unemployment rates of Māori were reduced to those of non-Māori. Some of these new jobs could have substantial co-benefits eg, additional tree planting on farmed hill country has benefits in terms of carbon sequestration, enhanced biodiversity, erosion prevention, flood prevention and improved water quality.

To conclude, this modeling work suggests that unemployment due to the COVID-19 pandemic could cause significant health loss, additional health system costs and exacerbate inequities from CVD in this high-income country. Prevention measures should be considered by governments to reduce this risk, including additional job creation programs and measures directed towards CVD prevention (eg, enhanced progress towards a smokefree country and reducing the hazardous processed food environment).

## Supporting information

S1 Table Health loss (in HALYs) results for the equity scenario (scenario analysis #A) (3% discount rate for the remaining life of the NZ population alive in 2011) for various unemployment scenarios. (DOCX)

S2 Table Health loss (in HALYs) results for the base case (unemployment persists for 10 years, scenario analysis #B) for various unemployment scenarios. (DOCX)

S3 Table Health loss (in HALYs) results for the base case (CVD incidence trend level-off, scenario analysis #C) for various unemployment scenarios. (DOCX)

S4 Table Health loss (in HALYs) results for the base case (0% discount rate, sensitivity analysis #D) for various unemployment scenarios. (DOCX)

S5 Table Health loss (in HALYs) results for the base case (6% discount rate, sensitivity analysis #E) for various unemployment scenarios. (DOCX)
