# Supplementary material for: Potential impact of COVID-19 related unemployment on increased cardiovascular disease in a high-income country: Modeling health loss, cost and equity
Source: PLoS One. 2021 May 27;16(5):e0246053. doi: 10.1371/journal.pone.0246053 (PMC8159004; doi:10.1371/journal.pone.0246053)
Supplement: S2 Table — (DOCX) [file pone.0246053.s002.docx]

**S2 Table Health loss (in HALYs) results for the base case (unemployment persists for 10 years, Scenario analysis #B) for various unemployment scenarios**

| **Groups** | **Change in HALYs** | | | | **Change in HALYs per 1000 capita** | | | | **Equity: Māori/Non-Māori HALYs per capita** |
| --- | --- | --- | --- | --- | --- | --- | --- | --- | --- |
|  | Base case | Early recovery in trade and services | Extended border control | Resurgence in community transmission | Base case | Early recovery in trade and services | Extended border control | Resurgence in community transmission | Base case |
| All ethnic groups, both sexes, all age-groups | -57,100 | -44,300 | -69,100 | -73,300 | -33.3 | -25.8 | -40.3 | -42.7 | … |
| 5 years into future | -3,100 | -2,510 | -3,530 | -4,040 | -1.8 | -1.5 | -2.1 | -2.4 | … |
| 10 years into future | -10,800 | -8,470 | -12,890 | -13,970 | -6.3 | -4.9 | -7.5 | -8.1 | … |
| 20 years into future | -29,600 | -23,000 | -35,800 | -38,100 | -17.3 | -13.4 | -20.9 | -22.2 | … |
| Māori, both sexes, all age-groups | -18,700 | -14,500 | -22,700 | -24,000 | -94.2 | -73.0 | -114.3 | -120.8 | 3.7 |
| Non-Māori, both sexes, all age-groups | -38,300 | -29,800 | -46,400 | -49,300 | -25.3 | -19.7 | -30.6 | -32.5 | … |
| Māori Men, all age-groups | -17,260 | -13,470 | -20,760 | -22,090 | -185.4 | -144.7 | -223.0 | -237.3 | 3.9 |
| Non-Māori Men, all age-groups | -34,900 | -27,200 | -42,000 | -44,800 | -47.4 | -36.9 | -57.0 | -60.8 | … |
| Māori Women, all age-groups | -1,480 | -1,050 | -1,980 | -1,920 | -14.0 | -10.0 | -18.8 | -18.2 | 3.2 |
| Non-Māori Women, all age-groups | -3,430 | -2,560 | -4,350 | -4,450 | -4.4 | -3.3 | -5.6 | -5.7 | … |
| Māori Men 35-44 | -7,980 | -6,260 | -9,550 | -10,200 | -208.4 | -163.4 | -249.3 | -266.3 | 4.3 |
| Māori Men 45-54 | -6,720 | -5,180 | -8,210 | -8,600 | -198.8 | -153.3 | -242.9 | -254.4 | 3.7 |
| Māori Men 55-64 | -2,560 | -2,030 | -2,990 | -3,290 | -121.9 | -96.7 | -142.4 | -156.7 | 3.2 |
| Non-Māori Men 35-44 | -12,050 | -9,430 | -14,450 | -15,440 | -48.4 | -37.9 | -58.0 | -62.0 | … |
| Non-Māori Men 45-54 | -14,350 | -11,020 | -17,630 | -18,430 | -54.0 | -41.4 | -66.3 | -69.3 | … |
| Non-Māori Men 55-64 | -8,500 | -6,740 | -9,940 | -10,980 | -38.4 | -30.4 | -44.9 | -49.6 | … |
| Māori Women 35-44 | 999 | 844 | 1,073 | 1,274 | 22.7 | 19.1 | 24.3 | 28.9 | 7.2 |
| Māori Women 45-54 | -1,378 | -1,058 | -1,690 | -1,771 | -36.1 | -27.7 | -44.2 | -46.4 | 5.6 |
| Māori Women 55-64 | -1,098 | -834 | -1,358 | -1,424 | -47.3 | -35.9 | -58.5 | -61.4 | 4.3 |
| Non-Māori Women 35-44 | 853 | 726 | 905 | 1,088 | 3.1 | 2.7 | 3.3 | 4.0 | … |
| Non-Māori Women 45-54 | -1,798 | -1,379 | -2,214 | -2,311 | -6.4 | -4.9 | -7.9 | -8.3 | … |
| Non-Māori Women 55-64 | -2,490 | -1,900 | -3,040 | -3,230 | -10.9 | -8.3 | -13.3 | -14.1 | … |

***Notes****: All analyses in this table were implemented without uncertainty. Furthermore, please refer to the Additional scenarios and sensitivity analyses in the Methods section for changes in assumptions compared to the base-case analysis. The equity ratio for Māori/Non-Māori HALYs lost per capita was very similar for all scenarios.*
